# Preventive drugs for Huntington’s disease: A choice-based conjoint survey of patient preferences

**DOI:** 10.1017/cts.2022.372

**Published:** 2022-03-01

**Authors:** Marcus C. Parrish, Andrea Hanson-Kahn, V. Srinivasan, Kevin V. Grimes

**Affiliations:** 1 SPARK Translational Research Program, Stanford University School of Medicine, Stanford, CA, USA; 2 Department of Chemical and Systems Biology, Stanford University School of Medicine, Stanford, CA, USA; 3 Department of Genetics, Stanford University School of Medicine, Stanford, CA, USA; 4 Stanford University Graduate School of Business, Stanford, CA, USA

**Keywords:** Huntington’s disease, patient preferences, choice-based conjoint survey, preventive treatment, regulatory policy

## Abstract

**Introduction:**

This research examined the perspective of the Huntington’s disease (HD) community regarding the use of predictive biomarkers as endpoints for regulatory approval of therapeutics to prevent or delay the onset of clinical HD in asymptomatic mutation carriers.

**Methods:**

An online, choice-based conjoint survey was shared with HD community members including untested at-risk individuals, presymptomatic mutation carriers, and symptomatic individuals. Across 15 scenarios, participants chose among two proposed therapies with differing degrees of biomarker improvement and side effects or a third option of no treatment.

**Results:**

Two hundred and thirty-eight responses were received. Attributes reflecting biomarker efficacy (e.g., prevention of brain atrophy on magnetic resonance imaging, reduced mutant huntingtin, or reduced inflammation biomarkers) had 3- to 7-fold greater importance than attributes representing side effects (e.g., increased risk of heart disease, cancer, and stroke over 20 years) and were more influential in directing choice of treatments. Reduction in mutant huntingtin protein was the most valued attribute overall. Multinomial logit model simulations based on survey responses demonstrated high interest among respondents (87–99% of the population) for drugs that might prevent or delay HD solely based upon biomarker evidence, even at the risk of serious side effects.

**Conclusion:**

These results indicate a strong desire among members of the HD community for preventive therapeutics and a willingness to accept significant side effects, even before the drug has been shown to definitively delay disease onset if the drug improves biomarker evidence of HD progression. Preferences of the HD community should inform regulatory policies for approving preventive therapies.

## Introduction

Huntington’s disease (HD) is an autosomal dominant, progressive, neurodegenerative disease that affects approximately 30,000 people in the USA. The disease is caused by a CAG trinucleotide repeat expansion in the huntingtin gene (*HTT*) with full disease penetrance when >39 repeats are present and partial penetrance with 36–39 repeats [1]. Thus, individuals who will develop clinical HD can be identified prior to symptom onset by genetic testing that measures the number of *HTT* CAG repeats.

While clinically manifest disease is typically diagnosed when motor symptoms appear around middle age, the definitive diagnosis may be preceded by more subtle symptoms including changes in mood, behavior, and cognition. Predicted age at onset of motor symptoms can be estimated using a formula that incorporates current age and number of CAG repeats [1]. Importantly, biomarkers thought to correlate with disease activity show progressive changes during the years and decades prior to clinical diagnosis [2,3]. High quality cross-sectional and longitudinal studies in premanifest mutation carriers show progressive regional brain atrophy on volumetric magnetic resonance imaging (MRI) studies, elevations in mutant huntingtin protein levels in plasma and cerebrospinal fluid (CSF), increased neurofilament light chain levels in plasma and CSF, elevated YKL-40 levels in CSF, and elevated plasma inflammatory markers such as IL-6 when compared to age-matched controls [4–17]. Moreover, these biomarkers worsen as the disease progresses to overt symptoms [2,18].

By the time of definitive diagnosis, MRI demonstrates a mean volumetric loss of 27% in both the caudate and putamen with an annualized rate of loss up to 4% and 3%, respectively, starting as early as 15 years prior to onset of motor symptoms [19,20]. Based on HD’s natural history, presymptomatic carriers could have an extended treatment window for preventive treatments before symptom onset.

Regulatory approval of new drugs typically requires demonstration of both safety and improvement in clinical endpoints. The US Food and Drug Administration (FDA) instituted an Accelerated Approval Program to allow earlier approval of drugs that address a serious unmet medical need based on improvement of a surrogate endpoint that is thought to predict clinical benefit [21]. Using surrogate endpoints can substantially shorten the time to FDA approval, but the drug company is required to conduct further studies to confirm the anticipated clinical benefit. In its guidance for development of therapeutics for early Alzheimer’s disease (AD) and amyotrophic lateral sclerosis, FDA encourages the development and use of surrogate biomarkers that will predict clinical benefit [22,23].

The use of the Accelerated Approval Program is particularly well suited for approval of drugs that might prevent or delay disease progression in presymptomatic HD patients for the following reasons: 1) the disease has 100% penetrance in individuals with >39 CAG repeats; 2) the age of clinical diagnosis can be approximated based on age and number of CAG repeats, thereby allowing treatment to be instituted prior to onset of motors symptoms at a time when surrogate biomarkers are known to diverge from the control population; and 3) the natural history of brain regional volumetric loss and CSF/serum biomarker measurements is well established and these surrogate biomarkers are thought to predict disease progression.

While the HD community has a strong interest in interventions that may prevent symptomatic disease, there has been little investigation into the views of at-risk, presymptomatic, and symptomatic HD individuals regarding their willingness to take preventive medications solely based upon improvement in biomarkers of disease activity before the drug has been shown to definitively delay symptom onset. In June 2020, FDA issued a final guidance document on Patient-Focused Drug Development with the goal of including patient experiences and preferences with respect to treatment of a disease [24]. One approach to investigate the patient perspective is through a choice-based conjoint (CBC) experiment, a survey method designed to measure the preferences of patients and the tradeoffs they would tolerate in a treatment [25]. Originally developed for consumer marketing purposes, medical researchers have used this method to analyze patient preferences in numerous disease areas including AD, multiple sclerosis, and exercise research [26–28].

Here, we report the results of a CBC study with the HD community. We investigated participants’ willingness to take hypothetical preventive therapies with various side effect profiles that demonstrated different levels of improvement in several biomarkers thought to correlate with disease activity.

## Methods

### Standard Protocol Approvals, Registrations, and Patient Consents

The study was approved by the Stanford University Institutional Review Board (Protocol #45684). All participants provided their fully informed written consent.

### Study Population

We recruited individuals from the HD community who were symptomatic with HD, presymptomatic mutation carriers, or untested and at-risk for HD based on family history. All participants who completed the study stated that they were 18 years or older, that they had a parent with HD, and that they had not tested negative for the mutant huntingtin gene. Participants were recruited through the Huntington’s Disease Society of America (HDSA) website, the Huntington’s Disease Youth Organization, and a series of paid advertisements on Facebook targeting those in the HD community. The advertisements were primarily shown to individuals over 18 years in age who displayed an interest in HD. Individuals who clicked the link were brought to a website displaying the consent document which outlined the purpose of the study and the potential risks and benefits of participation. Participants who completed every question in the survey received a $25 gift card to Amazon.com as an incentive. Participants whose survey durations were in the fastest quintile of respondents (<180 seconds) were excluded from analysis to screen out potential participants completing the survey multiple times. Survey responses were anonymous and not linked to any identifiable personal information. Contact information for the dispensation of the gift card was entered in a separate encrypted, password-protected database and not linked to participant responses.

Our target sample size was 150 respondents at-risk of developing HD and no more than 300 respondents in total. The survey was live from April 24, 2019, to May 24, 2019. We decided to end recruitment at 238 individuals due to successful recruitment of 156 at-risk participants. Similar sample sizes have been utilized in most published discreet choice studies [27,29,30].

### Choice of Attributes and Levels

Attributes that might suggest therapeutic efficacy were determined through a comprehensive literature search of biomarkers in the presymptomatic HD population. Biomarkers were selected that appeared to show a significant difference between gene negative and presymptomatic individuals and were investigated in multiple studies [2,4–8,31–33]. The three attributes representing therapeutic efficacy selected were reduction in mutant huntingtin protein, reduction in inflammatory markers, and reduction in brain shrinkage on imaging.

Attributes and levels representing potential side effects in preventive therapeutics were selected following a small focus group and a comprehensive search of the most common adverse events observed in HD clinical trials with drug interventions listed on clinicaltrials.gov. The focus group centered on discussing participant interest in preventive treatments, their knowledge and views of biomarkers, and the side effects they would be willing to tolerate. The three participants were recruited from San Francisco Bay Area HD clinics and support groups. Participants were untested and at-risk of HD based on family history and ranged in age from mid-20s to mid-50s. Race and ethnicity information were not collected. We selected three side effects that were either commonly listed in HD clinical trials or highlighted in the focus group as being either unacceptable or acceptable. The side effect attributes were an increased risk of headaches, stomach and sleep problems; an increased risk of anxiety, depression, and suicidal thoughts; and an increased risk of heart disease, cancer, and stroke over 20 years. The six attributes and levels used in the survey are found in Table 1.

**Table 1.** Treatment attributes and levels used by participants in the choice-based conjoint analysis

Attribute	Level
Reduction of mutant huntingtin protein	No improvement
	50% improvement
	100% improvement
Reduction of inflammation markers	No improvement
	50% improvement
	100% improvement
Reduction of brain shrinkage	No improvement
	50% improvement
	100% improvement
Risk of developing headaches, stomach, or sleep problems	1 out of 100 people affected
	10 out of 100 people affected
	20 out of 100 people affected
Risk of developing anxiety, depression, or suicidal thoughts	1 out of 100 people affected
	5 out of 100 people affected
	10 out of 100 people affected
Risk of developing cancer, heart disease, or stroke over 20 years	1 out of 1000 people affected
	1 out of 100 people affected
	5 out of 100 people affected

### Survey Design

The cross-sectional survey was conducted on the survey platform, Sawtooth Lighthouse Studio. The survey consisted of three distinct sections: demographic questions, HD education, and the CBC. Seven multiple choice demographic questions assessed participants’ gender, age, whether a participant’s parent has/had HD, whether the participant had tested for the mutant huntingtin gene, and whether they had tested positive. We asked about the device used to access the survey and required that participants use a desktop or laptop computer for optimal results. Respondents who were gene-negative, not at-risk for HD, or using a mobile device were excluded from the study.

In the second section of the survey, we briefly described HD, the symptoms that occur, and the genetic nature of the disease. In addition, we informed the participant about biomarker changes that have been observed to occur before symptom onset in HD. Finally, we outlined the third part of the survey where they were to assume to be at-risk for HD and had yet to show symptoms.

**Fig. 1.**
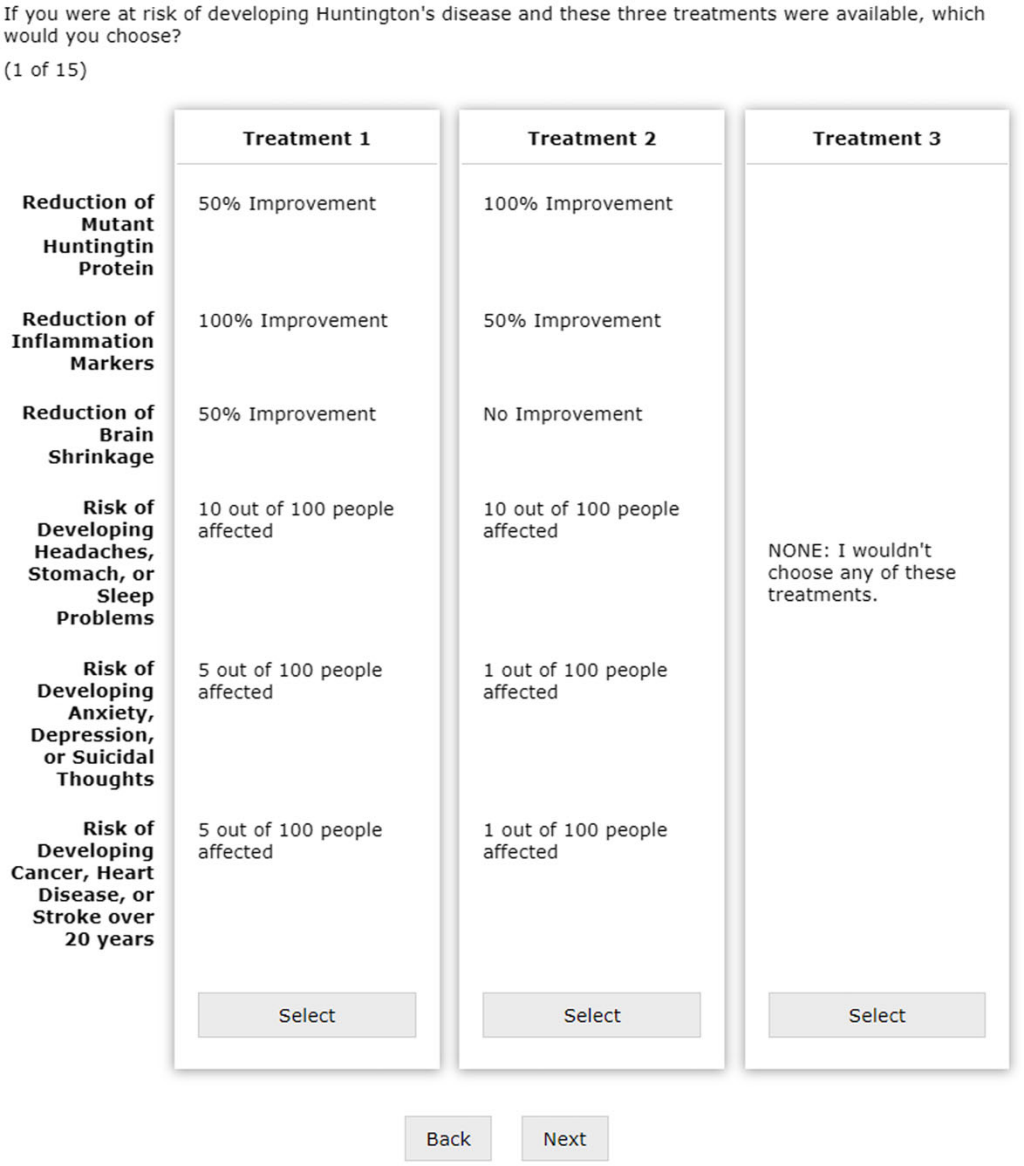
Sample choice set for choice-based conjoint analysis.

In the third and final section, participants were asked to choose between a pair of preventive therapeutics for HD. Figure 1 shows an example question. Each potential treatment in the choice task displayed the six attributes found in Table 1 and a randomly generated level for that attribute as developed by the conjoint analysis program Sawtooth Software [14]. The participant also had a third null option indicating that they would take neither treatment. Prior to initiating the CBC, participants were informed that no drug in the study directly represented any drug currently under study. Participants completed 15 choice tasks and were subsequently directed to a separate form to receive an Amazon gift card. The survey was pretested with individuals in the HD community for clarity and comprehension before administration. The full survey including a sample CBC section can be found in the Supplementary Material.

### Statistical Analysis

The CBC data were analyzed through the commonly used Hierarchical Bayes’ logit model [34]. The CBC software package from Sawtooth Software Inc. was used to analyze the data and to generate a model for simulations. All the parameters were specified to be normally distributed random parameters. No weighting was used to adjust for non-representativeness of the sample.

### Data Availability

Anonymized data relevant to this study will be made available by request from any qualified investigator pending appropriate Institutional Review Board approvals.

## Results

### Demographics

Eight hundred and sixty-seven visitors started the web-based survey. Five hundred and two visitors were screened out from the study due to demographics (e.g., age) or use of a mobile device and 82 did not complete the entire survey. Non-unique completes were determined by the total elapsed time of the survey; the 45 individuals who completed the survey in less than 180 seconds were deemed as non-unique and excluded from analyses. Two hundred and thirty-eight individuals successfully completed the web-based survey over the 1-month time period and were analyzed. The survey has a response rate of 27% defined as the number analyzed completes divided by the number of survey starts.

Demographics of the participant population can be found in Table 2. The majority were untested, at-risk individuals (UAR) (*N* = 156; 66%). The remainder comprised of self-reported presymptomatic mutation carriers (PSx) (*N* = 37; 15%) and symptomatic individuals (Sx) (*N* = 45; 19%). Given the low rate of early testing for the huntingtin gene and the advertisements’ focus on preventive medications, a high prevalence of UAR in the participant population was expected [35]. Participants’ racial and ethnic backgrounds were not collected in the study.

**Table 2.** Participant characteristics (N = 238)

		Untested, at-risk	Presymptomatic	Symptomatic	Total
		*N* = 156	*N* = 37	*N* = 45	*N* = 238
Sample size		66%	16%	19%	100%
Gender	Male	14%	38%	33%	21%
	Female	86%	62%	64%	78%
	Other	0%	0%	2%	0%
Age	18–29	44%	30%	18%	37%
	30–39	28%	49%	36%	33%
	40–49	16%	16%	18%	16%
	50–59	10%	3%	20%	11%
	60–69	1%	3%	7%	3%
	70+	0%	0%	2%	0%

Interestingly, the participant population had a gender imbalance with 78% of the participants identifying as female. This imbalance was observed across the three patient subclasses. The use of Facebook as the primary marketing method likely contributed to the uneven demographic [36]. Additional analyses have indicated no significant differences in the responses of the survey between men and women.

Overall, the age range of the population trended younger with a sizable percentage of the respondents between the ages of 18 and 39. The UAR and PSx populations contributed heavily to this breakdown with 72% and 79% of participants in the 18-to-39-year age range, respectively. The Sx population contained the majority of older participants with 47% of their population over the age of 40. Given the delay of symptom onset in HD, the increased age of Sx participants was expected.

### Biomarker attributes displayed significantly higher preference values compared to side effect attributes

**Fig. 2.**
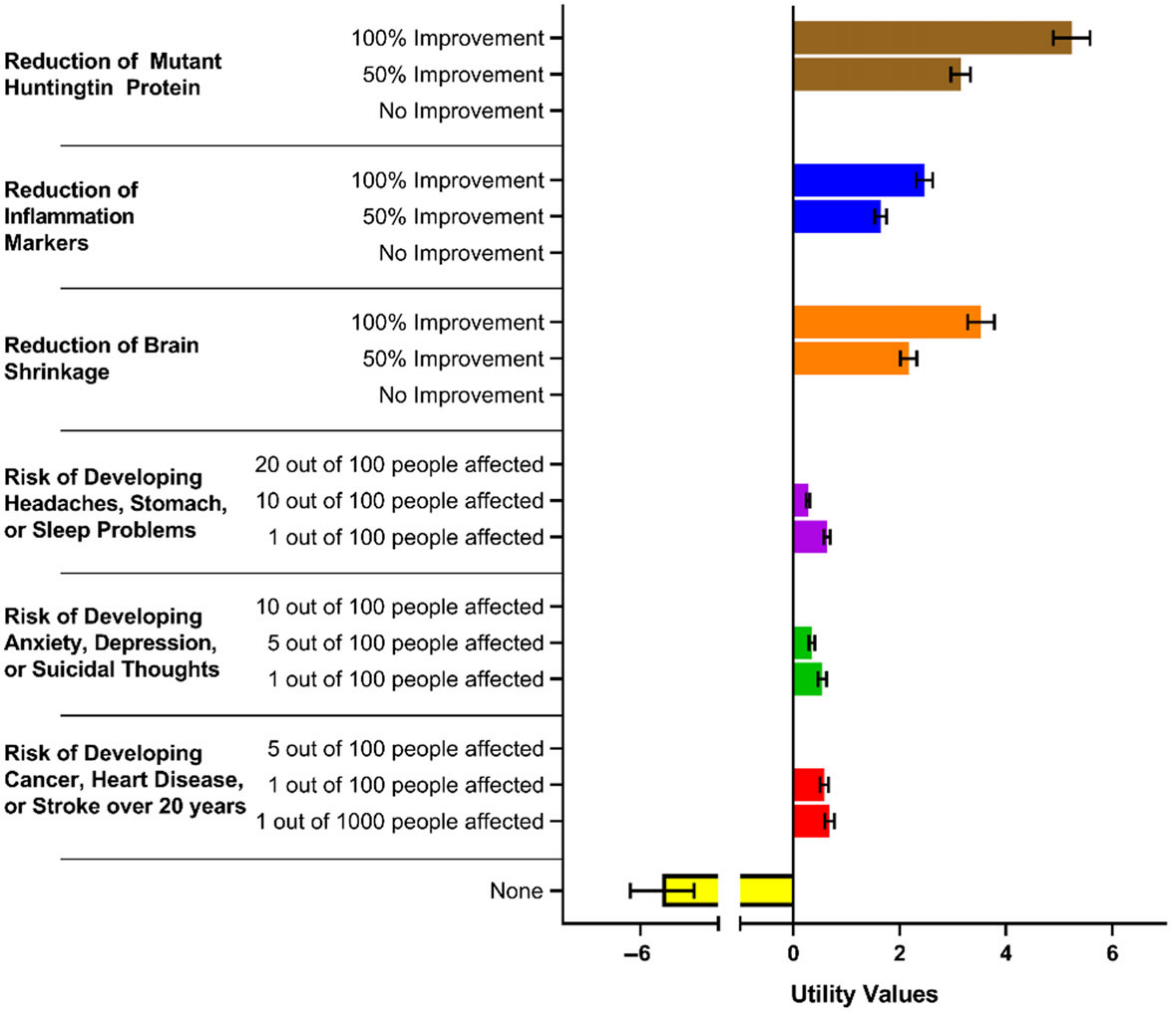
Average utility values for attributes and levels (*N* = 238). Error bars represent 95% confidence interval.

Table 3 and Fig. 2 display the attribute preferences of the participants calculated through a hierarchical Bayes’ model. Values normalized to 0 and 95% confidence intervals are displayed. Higher numbers represent an increased preference by the participants for the specific attribute. Within each attribute, the most beneficial level (e.g., 100% improvement in reduction of mutant huntingtin protein) had significantly greater values than the least beneficial levels (e.g., no improvement in reduction of mutant huntingtin protein). Thus, the most beneficial levels were preferred to the less beneficial levels. Moreover, the three attributes suggesting potential biomarkers of therapeutic efficacy (Reduction of Mutant Huntingtin Protein, Reduction of Inflammation Markers, Reduction of Brain Shrinkage) were considered more important than the three attributes representing side effect risk. Finally, analysis suggests a strongly negative interest in the “None” option representing no treatment. The “None” attribute had a significantly lower utility value than all other attributes.

**Table 3.** Results of hierarchical Bayes model – attribute preferences (N = 238)

Attribute	Level	Utility^*^	95% CI^**^	*p*-value^‡^
Reduction of mutant huntingtin protein	No improvement	Set to 0		
	50% improvement	3.15	2.96, 3.33	<0.001
	100% improvement	5.24	4.89, 5.58	<0.001
Reduction of inflammation markers	No improvement	Set to 0		
	50% improvement	1.64	1.53, 1.75	<0.001
	100% improvement	2.47	2.31, 2.62	<0.001
Reduction of brain shrinkage	No improvement	Set to 0		
	50% improvement	2.17	2.01, 2.32	<0.001
	100% improvement	3.53	3.28, 3.78	<0.001
Risk of developing headaches, stomach, or sleep problems	1 out of 100 people affected	0.63	0.57, 0.69	<0.001
	10 out of 100 people affected	0.28	0.24, 0.31	<0.001
	20 out of 100 people affected	Set to 0		
Risk of developing anxiety, depression, or suicidal thoughts	1 out of 100 people affected	0.54	0.46, 0.62	<0.001
	5 out of 100 people affected	0.35	0.29, 0.40	<0.001
	10 out of 100 people affected	Set to 0		
Risk of developing cancer, heart disease, or stroke over 20 years	1 out of 1000 people affected	0.68	0.59, 0.77	<0.001
	1 out of 100 people affected	0.58	0.50, 0.66	<0.001
	5 out of 100 people affected	Set to 0		
None		−5.72	−6.13, −5.31	

* Utility indicates the relative attractiveness of a product with that attribute.

** CI = Confidence Interval.

‡ Paired Student’s *t*-test comparison of that level with the worst level of that attribute.

### Minimal difference observed in the importance of attributes among UAR, PSx, and Sx participants

**Fig. 3.**
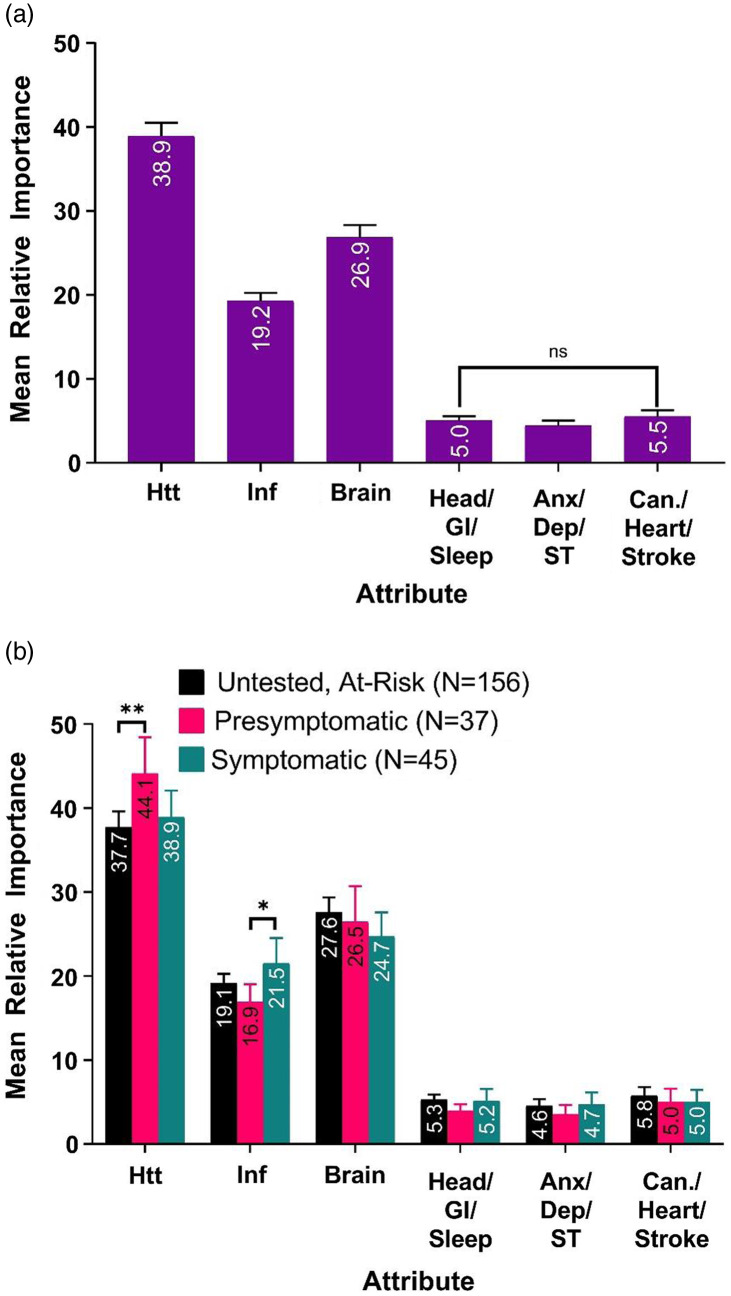
Average importance of attributes. (*A*) Average importance of attributes overall (*N* = 238). All differences are statistically significant unless otherwise indicated (*p* < 0.05, paired sample t-test). ns: not statistically significant. Error bars represent 95% confidence Interval. (*B*) Average importance of attributes segmented by patient subgroup. *p < 0.05, two-group t-test; **p < 0.01 error bars represent 95% confidence intervals. Htt: Reduction of Mutant Huntingtin Protein; Inf: Reduction of Inflammatory Markers; Brain: Reduction of Brain Shrinkage; Head/GI/Sleep: Risk of Developing Headaches, Stomach, and Sleep Problems; Anx/Dep/ST: Risk of Developing Anxiety, Depression, or Suicidal Thoughts; Can/Heart/Stroke: Risk of Developing Cancer, Heart Disease, or Stroke over 20 years.

The importance of an attribute is calculated by subtracting the lowest utility values from the highest utility values of an attribute and dividing by the sum of the differences across all attributes. Figure 3A shows the average importance of the six tested attributes in the CBC. Reduction of Mutant Huntingtin protein was the most important attribute (38.9%, CI: 37.3–40.5%) followed by Reduction of Brain Shrinkage (26.9%, CI: 25.4–28.4%) and Reduction of Inflammatory Markers (19.2%, CI: 18.2–20.2%). All of these beneficial markers were 3–7× more important than the attributes related to side effects. Thus, it appears that the potential benefit given by a preventive drug drives the selection of a treatment more than the potential risk.

In Fig. 3B, we analyzed the importance of attributes within the three participant subgroups. There is a significant difference in the importance of the Reduction of Mutant Huntingtin Protein between PSx and UAR individuals (*p* < 0.01; PSx CI: 39.8–48.4; UAR CI: 35.8–39.6), and a significant difference between the importance of the Reduction of Inflammatory Markers between PSx and Sx (*p* < 0.05; PSx CI: 14.8–19; Sx CI: 18.5–24.5). Nevertheless, the overall trend of attribute importance amongst the subgroups was surprisingly consistent. Participants were most influenced by treatments that would reduce huntingtin protein, followed by those that reduce brain shrinkage and those that reduce inflammation in the brain. The three attributes related to side effect were effectively indistinguishable from each other in terms of their low importance.

### The HD community is willing to tolerate high side effects for potential benefit

From our analysis of the CBC, we can use the multinomial logit model in the Sawtooth Software Inc. and simulate the HD population’s interest in various preventive HD therapeutics (i.e., choosing a specific treatment over no treatment). Table 4 outlines attributes of four potential therapies and the predicted community uptake of the treatment. Drug A, a hypothetical treatment that demonstrates very high efficacy in all three markers and the lowest possible risk of side effects, had the highest predicted interest with 99% of the HD community interested in the drug. Drug B, which has similar efficacy to Drug A, but the highest risk of side effects, was also predicted to have a high interest of 96%. If a preventive has only 50% efficacy in biomarkers and minimal side effects, as in Drug C, then 95% of the HD community is predicted to be interested in the drug. Increasing the risk of the side effects in the therapeutic, as in Drug D, only reduced the predicted percentage to 87%. In every scenario, a very small percentage of the HD community is predicted to be interested in the “no treatment” option.

**Table 4.** Simulation of the Huntington’s disease (HD) community’s uptake of drugs with varying attributes

	Reduction of: mutant huntingtin protein, inflammation markers, and brain shrinkage	Risk of developing headaches, stomach, or sleep problems	Risk of developing anxiety, depression, or suicidal thoughts	Risk of developing cancer, heart disease, or stroke over 20 years	Predicted HD community uptake of drug vs. no treatment (%)
**Drug A:** 100% biomarker benefit, low risk	100% improvement	1 out of 100 people affected	1 out of 100 people affected	1 out of 1000 people affected	99%
**Drug B:** 100% biomarker benefit, high risk	100% improvement	20 out of 100 people affected	10 out of 100 people affected	5 out of 100 people affected	96%
**Drug C:** 50% biomarker benefit, low risk	50% improvement	1 out of 100 people affected	1 out of 100 people affected	1 out of 1000 people affected	95%
**Drug D:** 50% biomarker benefit, high risk	50% improvement	20 out of 100 people affected	10 out of 100 people affected	5 out of 100 people affected	87%

## Discussion

In this study, we investigated the HD community’s views towards preventive medications and analyzed the side effects they would tolerate for potential therapeutic benefit as assessed by biomarkers. By performing a CBC experiment, we show that there is strong interest from the community in HD preventive treatments. For instance, the “None” attribute listed in Fig. 2, which indicates interest in forgoing preventive treatment, had a significantly lower utility value than all other attributes. Moreover, the predicted low interest in the no treatment option in every scenario in Table 4 suggests that the HD community is willing to try many medications that have some possibility of efficacy. This observation is supported by anecdotal evidence by the FDA. In their 2016 Report: *The Voice of the Patient: Huntington’s Disease*, the FDA wrote: “Outside of a cure, many participants wanted medication that could drastically slow progression of symptoms or delay the onset of symptoms.…One participant commented on the need to identify or develop therapies that ‘can be given whether you’re at risk or you are gene-positive, very, very early, well before onset’” [37].

When assessing preventive therapeutic options, participants put a great deal more weight on the efficacy measures than the potential side effects. Each of the biomarker measures had a three- to seven-fold greater importance than the side effect risks (Fig. 3), further supporting a strong tolerance for side effects for potential benefit. Reduction of mutant huntingtin protein was the most valued attribute, having the greatest influence on participants’ decision. Interestingly, presymptomatic individuals valued mutant huntingtin protein more than untested at-risk individuals by a small but statistically significant amount. The slight difference in preference is likely due to presymptomatic individuals having increased knowledge of HD research. Mutant huntingtin protein has been an important topic of the research community for years and previous research has observed that the presymptomatic community has a significantly higher level of knowledge of HD research compared to untested at-risk individuals [38]. While there is a slight difference in perception, both subgroups’ view of mHTT was significantly increased compared to other attributes. This result suggests despite minor differences between subgroups, the community has a strong understanding of the role that mutant huntingtin protein plays in HD and interest in it as a biomarker.

Furthermore, our survey demonstrates that the HD community has a high tolerance for risk of side effects. Increasing the risk of side effects from the lowest to the highest levels in highly effective drugs only reduced intention to take the therapeutic by 3% (Table 4 Drug A and B). Similarly, increasing the risk of side effects in mildly effective drugs only reduced interest in the drug by 8% (Table 4 Drug C and D).

Similar studies have been performed in other disease areas including rheumatoid arthritis, breast cancer, osteoporosis, and AD. Interestingly, in prior non-neurological studies, the participants display a low willingness to use a preventive therapeutic [39–41]. Additionally, these studies have shown that individuals have a high aversion to preventive treatments with any risk of side effects [42]. However, a high interest in preventive treatment and a high tolerance of side effect risk have been observed in AD [43,44]. This difference in preference may be due to the fear of dementia in older populations and the absence of disease-modifying therapies [45,46]. The lack of therapeutic alternatives may increase participant willingness to select a treatment with a high-risk profile. If a disease-modifying treatment becomes available, the preferences of the disease community may change in accordance with the drug’s efficacy and side effect profile. Previous research has also demonstrated that this current pro-treatment mindset can confound results with some participants illogically selecting treatments with no benefit and high side effect risk [43]. Additional investigations should be performed to further understand the HD community’s views on preventive medications over time, taking into consideration the community’s pro-treatment bias and other treatment attributes that are outside the scope of this study including the method of treatment administration, the durability of the treatment, and even higher side effect risk.

In addition, further analysis into the role of race and ethnicity on the HD community’s views on preventive treatments should be studied. While this investigation did not collect race or ethnicity information from respondents, we hypothesize most respondents to be those of Caucasian or African descent given the diagnostic frequency observed in previous research [47]. Deeper speculation into the differences in perception of preventive treatments between racial and ethnic groups is beyond the scope of this study; however, further research is needed to support racial equity in the HD community and the US healthcare system. By highlighting the importance of this demographic information in HD clinical studies, we can reduce the potential of race-based disparity in clinical trial enrollment observed in other neurodegenerative diseases [48,49].

This study has three primary limitations. First, the participants who completed the survey may not accurately represent the entire HD community. Individuals who are reluctant to complete online surveys on HD may have different views than those presented here. In addition, by focusing our recruitment strategies on those with a demonstrated interest in HD or the HDSA, our participant population may be biased against those less involved in the HD community. Thus, our analysis is best understood as a representation of more active members of the HD community.

Second, our simulation of interest in potential HD drugs only indicates an intention to take a preventive medication. Individuals may have a different response when actually offered the choice to start a therapeutic by their physician, and the current percentages may be overstated. As more evidence becomes available regarding biomarker performance and potential drug side effects, additional studies should be performed to provide further insights on the HD community’s preferences regarding preventive therapeutics.

Finally, while the survey does demonstrate participant tolerance for side effects based on their incidence, it does not include information on severity. The potential for associated disability would be an important factor in treatment discussions with patients and should be investigated in future studies. However, the relatively low importance of side effect attributes remains an informative finding given the diversity of attributes investigated. While headaches, stomach, and sleep problems can range from mildly inconvenient to severely debilitating, all grades of heart disease, stroke, and cancer are serious medical complications. The insight that a majority of patients are interested in these preventive treatments regardless of side effect attribute and the lack of discrimination between side effects further indicates the desire for preventive treatments in HD.

Preventive medications are highly desirable as a means to maintain health and prolong life in individuals at risk of developing symptomatic HD. The use of predictive biomarkers as clinical trial endpoints could accelerate the approval and clinical adoption of these drugs. This study indicates a strong desire among members of the HD community for preventive therapeutics and a willingness to accept significant side effects, even before the drug has been shown to definitively delay disease onset, if the drug improves biomarker evidence of HD progression. These preferences of the HD community should inform the development of new clinical and regulatory paradigms for advancing preventive therapies in HD.

## Supplementary material

For supplementary material accompanying this paper visit https://doi.org/10.1017/cts.2022.372. click here to view supplementary material
