# Supplementary material for: Preventive drugs for Huntington’s disease: A choice-based conjoint survey of patient preferences
Source: J Clin Transl Sci. 2022 Mar 1;6(1):e35. doi: 10.1017/cts.2022.372 (PMC9003635; doi:10.1017/cts.2022.372)
Supplement: Supplementary file 1 [file S2059866122003727sup001.pdf]

Supplementary material:

Full survey including IRB consent form and example choice-based conjoint section

Research Consent Form

Protocol Director: Marcus Parrish
Protocol Title: Survey of the Huntington's Disease Community's Views on Biomarkers and Preventive Drugs

STUDY INFORMATION

FOR QUESTIONS ABOUT THE STUDY, CONTACT: Marcus C. Parrish
269 Campus Dr.
Building CCSR Room 3145
Stanford, CA 95126
650-723-7833
HDstudy@stanford.edu

DESCRIPTION: You are invited to participate in a research study on preventive treatments in Huntington's Disease. The purpose of the study is to understand your preference for future treatments that may delay or prevent Huntington's Disease (HD) among individuals at-risk of developing HD. In addition, we would like to learn about the side effects, you would be willing to tolerate for the benefit. If you agree to participate, you will be asked to complete a brief survey and answer a series of questions to determine which treatments you prefer and the side effects you would be willing to tolerate to gain the benefit of preventing or delaying the progression of HD.

RISKS AND BENEFITS of participation is completing this survey. The only small risk is the potential of emotional distress from

learning more about the Huntington's disease process. We cannot and do not guarantee or promise that you will receive any benefits from this study. The potential benefit to society is knowledge gained regarding the HD community's views toward preventive treatments and their side effects.

TIME INVOLVEMENT: Your participation in this experiment will take approximately 15 minutes.

PAYMENTS: You will receive a $25 Amazon gift card as payment for your participation.

SURVEY DEVICE: This survey should only be taken on a laptop or a desktop. Please do NOT take this survey on a mobile device because the survey will not display correctly.

CONFIDENTIALITY: The data for this study will be kept anonymous, which would make it impossible for us to connect your responses with any of your identifying information.

PARTICIPANT'S RIGHTS: If you have read this form and have decided to participate in this project, please understand that your participation is voluntary and you have the right to withdraw your consent or discontinue participation at any time without penalty or loss of benefits to which you are otherwise entitled. You have the right to refuse to answer particular questions. The results of this research study may be presented at scientific or professional meeting or published in scientific journals. However, you identity will not be disclosed. Your alternative is not to participate. Your information and/or specimens will not be used or distributed for future research studies even if all identifying information is removed.

CONTACT INFORMATION: Questions, Concerns, or Complaints: If you have any questions, concerns or complaints about the research study, its procedures, or risks and benefits you ask the Protocol Director, Marcus Parrish. You may contact him now or later at HDStudy@stanford.edu or 650-723-7833.

Independent Contact: If you are not satisfied with how this study is being conducted, or if you have any concerns, complaints, or general questions about the research or your rights as a participant, please contact the Stanford Institutional Review Board (IRB) to speak to someone independent of the research team at (650)-723-5244 or toll free at 1-866-680-2906. You can also write to the Stanford IRB, Stanford University, 3000 El Camino Real, Five Palo Alto Square, 4th Floor, Palo Alto, CA, 94306.

Please print a copy of this page for your records. If you agree to participate in this survey, please click “Agree” to begin the survey.

- ○ Agree
- ○ Disagree

Next

Thank you very much for agreeing to participate in our survey on preventive treatments for Huntington’s disease. The goal of this survey is to determine your level of interest in a treatment that might prevent or delay progression to Huntington’s Disease in individuals who may be at risk of developing HD. In addition, we are eager to learn what side effects you would be willing to tolerate to gain the benefits.

To begin please answer a few demographic questions.

1. What is your gender?
    - ○ Male
    - ○ Female
    - ○ Other
2. How old are you?
    - ○ < 18 years old
    - ○ 18 - 29 years old
    - ○ 30 - 39 years old
    - ○ 40 - 49 years old
    - ○ 50 - 59 years old
    - ○ 60 - 69 years old
    - ○ >70 years old
3. Does or did one or both of your parents have Huntington’s disease?
    - ○ Yes
    - ○ No

Back
Next

4. Have you been tested for the Huntington’s Disease gene?
   - ○ Yes
   - ○ No

Back
Next

5. Are you positive for the Huntington’s Disease gene?
    - ○ Yes
    - ○ No

Back
Next

6. Have you begun to show symptoms?

- ○ Yes
- ○ No

If you have begun to show symptoms, you may prefer to complete the survey with the help of a caretaker.

Back
Next

On which device are you taking this survey?

- ○ Desktop
- ○ Laptop
- ○ Smartphone/Mobile Device

Back

Next

The following final phase of the survey is only fifteen screens long. At the end, you will be sent to a page to receive your $25 gift card.

Back

Next

Huntington’s disease (**HD**) is a genetic disease that leads to the loss of nerve cells in the brain causing multiple symptoms including:

- involuntary movement of limbs
- memory loss
- depression.

Every child of a parent with HD has a 50/50 chance of inheriting the expanded gene (**the huntingtin gene**) that causes HD. These people are considered “at-risk” of getting HD.

Back
Next

While the symptoms of HD often begin during middle age, HD can cause changes in the body years earlier. Studies have shown three primary changes in HD patients before symptoms occur:

1. A reduction in brain size
2. An increase in inflammation in the brain
3. An increase in huntingtin protein in the brain and blood. (Huntingtin protein is the product of the huntingtin gene which causes HD.)

Each of these primary changes have been shown to track with symptom development.

Back
Next

This survey supposes that you:

- Are at-risk of developing HD (your parents have had Huntington's Disease)
- Have yet to develop symptoms.

In this survey, assume that there are new treatments that could prevent HD. These treatments bear no relation to any real medication currently being used.

Suppose the treatments have only shown some effect on the three primary changes on the last page. They have not yet been proven to prevent HD. Moreover, the treatments have some risk of side effects.

On each of the following screens please select the treatment (out of two) you would most likely choose. You can also decide that you would take neither of the two treatments. Each of the choices will require you to balance potential benefits and short-term/long-term side effects.

Back
Next

Skip to:

If you were at risk of developing Huntington's disease and these three treatments were available, which would you choose?

(1 of 15)

	Treatment 1	Treatment 2	Treatment 3
**Reduction of Mutant Huntingtin Protein**	50% Improvement	No Improvement	NONE: I wouldn't choose any of these treatments.
**Reduction of Inflammation Markers**	100% Improvement	50% Improvement	
**Reduction of Brain Shrinkage**	100% Improvement	No Improvement	
**Risk of Developing Headaches, Stomach, or Sleep Problems**	1 out of 100 people affected	20 out of 100 people affected	
**Risk of Developing Anxiety, Depression, or Suicidal Thoughts**	1 out of 100 people affected	1 out of 100 people affected	
**Risk of Developing Cancer, Heart Disease, or Stroke over 20 years**	5 out of 100 people affected	1 out of 1000 people affected	
	Select	Select	Select

Back Next

0% 100%

Skip to:

If you were at risk of developing Huntington's disease and these three treatments were available, which would you choose?

(2 of 15)

	Treatment 1	Treatment 2	Treatment 3
**Reduction of Mutant Huntingtin Protein**	No Improvement	100% Improvement	NONE: I wouldn't choose any of these treatments.
**Reduction of Inflammation Markers**	50% Improvement	100% Improvement	
**Reduction of Brain Shrinkage**	No Improvement	100% Improvement	
**Risk of Developing Headaches, Stomach, or Sleep Problems**	20 out of 100 people affected	10 out of 100 people affected	
**Risk of Developing Anxiety, Depression, or Suicidal Thoughts**	10 out of 100 people affected	1 out of 100 people affected	
**Risk of Developing Cancer, Heart Disease, or Stroke over 20 years**	1 out of 1000 people affected	5 out of 100 people affected	
	Select	Select	Select

Back Next

0% 100%

Skip to:

If you were at risk of developing Huntington's disease and these three treatments were available, which would you choose?

(3 of 15)

	Treatment 1	Treatment 2	Treatment 3
**Reduction of Mutant Huntingtin Protein**	50% Improvement	100% Improvement	NONE: I wouldn't choose any of these treatments.
**Reduction of Inflammation Markers**	50% Improvement	100% Improvement	
**Reduction of Brain Shrinkage**	100% Improvement	50% Improvement	
**Risk of Developing Headaches, Stomach, or Sleep Problems**	1 out of 100 people affected	20 out of 100 people affected	
**Risk of Developing Anxiety, Depression, or Suicidal Thoughts**	5 out of 100 people affected	10 out of 100 people affected	
**Risk of Developing Cancer, Heart Disease, or Stroke over 20 years**	1 out of 100 people affected	1 out of 1000 people affected	
	Select	Select	Select

Back Next

0% 100%

Skip to:

If you were at risk of developing Huntington's disease and these three treatments were available, which would you choose?

(4 of 15)

	Treatment 1	Treatment 2	Treatment 3
**Reduction of Mutant Huntingtin Protein**	50% Improvement	No Improvement	NONE: I wouldn't choose any of these treatments.
**Reduction of Inflammation Markers**	No Improvement	No Improvement	
**Reduction of Brain Shrinkage**	50% Improvement	No Improvement	
**Risk of Developing Headaches, Stomach, or Sleep Problems**	1 out of 100 people affected	10 out of 100 people affected	
**Risk of Developing Anxiety, Depression, or Suicidal Thoughts**	10 out of 100 people affected	10 out of 100 people affected	
**Risk of Developing Cancer, Heart Disease, or Stroke over 20 years**	1 out of 100 people affected	1 out of 1000 people affected	
	Select	Select	Select

Back Next

0% 100%

Skip to:

If you were at risk of developing Huntington's disease and these three treatments were available, which would you choose?

(5 of 15)

	Treatment 1	Treatment 2	Treatment 3
**Reduction of Mutant Huntingtin Protein**	No Improvement	50% Improvement	NONE: I wouldn't choose any of these treatments.
**Reduction of Inflammation Markers**	100% Improvement	50% Improvement	
**Reduction of Brain Shrinkage**	50% Improvement	No Improvement	
**Risk of Developing Headaches, Stomach, or Sleep Problems**	1 out of 100 people affected	10 out of 100 people affected	
**Risk of Developing Anxiety, Depression, or Suicidal Thoughts**	5 out of 100 people affected	1 out of 100 people affected	
**Risk of Developing Cancer, Heart Disease, or Stroke over 20 years**	1 out of 100 people affected	1 out of 100 people affected	
	Select	Select	Select

Back Next

0% 100%

Skip to:

If you were at risk of developing Huntington's disease and these three treatments were available, which would you choose?

(6 of 15)

	Treatment 1	Treatment 2	Treatment 3
**Reduction of Mutant Huntingtin Protein**	100% Improvement	100% Improvement	NONE: I wouldn't choose any of these treatments.
**Reduction of Inflammation Markers**	No Improvement	50% Improvement	
**Reduction of Brain Shrinkage**	100% Improvement	No Improvement	
**Risk of Developing Headaches, Stomach, or Sleep Problems**	20 out of 100 people affected	10 out of 100 people affected	
**Risk of Developing Anxiety, Depression, or Suicidal Thoughts**	1 out of 100 people affected	5 out of 100 people affected	
**Risk of Developing Cancer, Heart Disease, or Stroke over 20 years**	5 out of 100 people affected	1 out of 1000 people affected	
	Select	Select	Select

Back Next

0% 100%

Skip to:

If you were at risk of developing Huntington's disease and these three treatments were available, which would you choose?

(7 of 15)

	Treatment 1	Treatment 2	Treatment 3
**Reduction of Mutant Huntingtin Protein**	50% Improvement	No Improvement	NONE: I wouldn't choose any of these treatments.
**Reduction of Inflammation Markers**	No Improvement	50% Improvement	
**Reduction of Brain Shrinkage**	100% Improvement	No Improvement	
**Risk of Developing Headaches, Stomach, or Sleep Problems**	20 out of 100 people affected	1 out of 100 people affected	
**Risk of Developing Anxiety, Depression, or Suicidal Thoughts**	10 out of 100 people affected	10 out of 100 people affected	
**Risk of Developing Cancer, Heart Disease, or Stroke over 20 years**	1 out of 1000 people affected	5 out of 100 people affected	
	Select	Select	Select

Back Next

0% 100%

Skip to:

If you were at risk of developing Huntington's disease and these three treatments were available, which would you choose?

(8 of 15)

	**Treatment 1**	**Treatment 2**	**Treatment 3**
**Reduction of Mutant Huntingtin Protein**	100% Improvement	No Improvement	NONE: I wouldn't choose any of these treatments.
**Reduction of Inflammation Markers**	100% Improvement	50% Improvement	
**Reduction of Brain Shrinkage**	50% Improvement	100% Improvement	
**Risk of Developing Headaches, Stomach, or Sleep Problems**	10 out of 100 people affected	1 out of 100 people affected	
**Risk of Developing Anxiety, Depression, or Suicidal Thoughts**	1 out of 100 people affected	5 out of 100 people affected	
**Risk of Developing Cancer, Heart Disease, or Stroke over 20 years**	1 out of 100 people affected	1 out of 1000 people affected	
	Select	Select	Select

Back Next

0% 100%

Skip to:

If you were at risk of developing Huntington's disease and these three treatments were available, which would you choose?

(9 of 15)

	**Treatment 1**	**Treatment 2**	**Treatment 3**
**Reduction of Mutant Huntingtin Protein**	100% Improvement	50% Improvement	NONE: I wouldn't choose any of these treatments.
**Reduction of Inflammation Markers**	No Improvement	100% Improvement	
**Reduction of Brain Shrinkage**	No Improvement	100% Improvement	
**Risk of Developing Headaches, Stomach, or Sleep Problems**	10 out of 100 people affected	20 out of 100 people affected	
**Risk of Developing Anxiety, Depression, or Suicidal Thoughts**	10 out of 100 people affected	5 out of 100 people affected	
**Risk of Developing Cancer, Heart Disease, or Stroke over 20 years**	5 out of 100 people affected	1 out of 1000 people affected	
	Select	Select	Select

Back Next

0% 100%

Skip to:

If you were at risk of developing Huntington's disease and these three treatments were available, which would you choose?

(10 of 15)

	Treatment 1	Treatment 2	Treatment 3
**Reduction of Mutant Huntingtin Protein**	No Improvement	50% Improvement	NONE: I wouldn't choose any of these treatments.
**Reduction of Inflammation Markers**	No Improvement	100% Improvement	
**Reduction of Brain Shrinkage**	50% Improvement	50% Improvement	
**Risk of Developing Headaches, Stomach, or Sleep Problems**	20 out of 100 people affected	10 out of 100 people affected	
**Risk of Developing Anxiety, Depression, or Suicidal Thoughts**	1 out of 100 people affected	5 out of 100 people affected	
**Risk of Developing Cancer, Heart Disease, or Stroke over 20 years**	1 out of 100 people affected	5 out of 100 people affected	
	Select	Select	Select

Back Next

0% 100%

Skip to:

If you were at risk of developing Huntington's disease and these three treatments were available, which would you choose?

(11 of 15)

	Treatment 1	Treatment 2	Treatment 3
**Reduction of Mutant Huntingtin Protein**	100% Improvement	50% Improvement	NONE: I wouldn't choose any of these treatments.
**Reduction of Inflammation Markers**	50% Improvement	100% Improvement	
**Reduction of Brain Shrinkage**	100% Improvement	No Improvement	
**Risk of Developing Headaches, Stomach, or Sleep Problems**	20 out of 100 people affected	1 out of 100 people affected	
**Risk of Developing Anxiety, Depression, or Suicidal Thoughts**	5 out of 100 people affected	1 out of 100 people affected	
**Risk of Developing Cancer, Heart Disease, or Stroke over 20 years**	1 out of 100 people affected	1 out of 100 people affected	
	Select	Select	Select

Back Next

0% 100%

Skip to:

If you were at risk of developing Huntington's disease and these three treatments were available, which would you choose?

(12 of 15)

	Treatment 1	Treatment 2	Treatment 3
**Reduction of Mutant Huntingtin Protein**	100% Improvement	No Improvement	NONE: I wouldn't choose any of these treatments.
**Reduction of Inflammation Markers**	No Improvement	50% Improvement	
**Reduction of Brain Shrinkage**	50% Improvement	No Improvement	
**Risk of Developing Headaches, Stomach, or Sleep Problems**	10 out of 100 people affected	1 out of 100 people affected	
**Risk of Developing Anxiety, Depression, or Suicidal Thoughts**	10 out of 100 people affected	1 out of 100 people affected	
**Risk of Developing Cancer, Heart Disease, or Stroke over 20 years**	5 out of 100 people affected	1 out of 1000 people affected	
	Select	Select	Select

Back Next

0% 100%

Skip to:

If you were at risk of developing Huntington's disease and these three treatments were available, which would you choose?

(13 of 15)

	Treatment 1	Treatment 2	Treatment 3
**Reduction of Mutant Huntingtin Protein**	No Improvement	100% Improvement	NONE: I wouldn't choose any of these treatments.
**Reduction of Inflammation Markers**	50% Improvement	No Improvement	
**Reduction of Brain Shrinkage**	No Improvement	100% Improvement	
**Risk of Developing Headaches, Stomach, or Sleep Problems**	20 out of 100 people affected	1 out of 100 people affected	
**Risk of Developing Anxiety, Depression, or Suicidal Thoughts**	5 out of 100 people affected	1 out of 100 people affected	
**Risk of Developing Cancer, Heart Disease, or Stroke over 20 years**	5 out of 100 people affected	1 out of 100 people affected	
	Select	Select	Select

Back Next

0% 100%

Skip to:

If you were at risk of developing Huntington's disease and these three treatments were available, which would you choose?

(14 of 15)

	Treatment 1	Treatment 2	Treatment 3
**Reduction of Mutant Huntingtin Protein**	No Improvement	50% Improvement	NONE: I wouldn't choose any of these treatments.
**Reduction of Inflammation Markers**	100% Improvement	No Improvement	
**Reduction of Brain Shrinkage**	50% Improvement	100% Improvement	
**Risk of Developing Headaches, Stomach, or Sleep Problems**	1 out of 100 people affected	10 out of 100 people affected	
**Risk of Developing Anxiety, Depression, or Suicidal Thoughts**	10 out of 100 people affected	10 out of 100 people affected	
**Risk of Developing Cancer, Heart Disease, or Stroke over 20 years**	1 out of 1000 people affected	1 out of 100 people affected	
	Select	Select	Select

Back Next

0% 100%

Skip to:

If you were at risk of developing Huntington's disease and these three treatments were available, which would you choose?

(15 of 15)

	Treatment 1	Treatment 2	Treatment 3
**Reduction of Mutant Huntingtin Protein**	50% Improvement	100% Improvement	NONE: I wouldn't choose any of these treatments.
**Reduction of Inflammation Markers**	100% Improvement	50% Improvement	
**Reduction of Brain Shrinkage**	No Improvement	50% Improvement	
**Risk of Developing Headaches, Stomach, or Sleep Problems**	10 out of 100 people affected	20 out of 100 people affected	
**Risk of Developing Anxiety, Depression, or Suicidal Thoughts**	1 out of 100 people affected	5 out of 100 people affected	
**Risk of Developing Cancer, Heart Disease, or Stroke over 20 years**	1 out of 1000 people affected	5 out of 100 people affected	
	Select	Select	Select

Back Next

0% 100%

Thank You

Thank you for completing our survey. If you would like to receive a $25 Amazon gift card, please click <u>here.</u>

Stanford | MEDICINE
